# Comparative proteomic analysis of dental pulp from supernumerary and normal permanent teeth

**DOI:** 10.1007/s00784-024-05698-z

**Published:** 2024-05-17

**Authors:** Kritkamon Lertruangpanya, Sittiruk Roytrakul, Rudee Surarit, Sivaporn Horsophonphong

**Affiliations:** 1 Department of Pediatric Dentistry, Faculty of Dentistry, Mahidol University, 6 Yothi Road, Ratchathewi, Bangkok, Thailand; 2 Functional Proteomics Technology Laboratory, National Center for Genetic Engineering and Biotechnology, National Science and Technology Development Agency, Pathumthani, Thailand; 3 Department of Oral Biology, Faculty of Dentistry, Mahidol University, Bangkok, Thailand; 4 Faculty of Dentistry, Siam University, Bangkok, Thailand

**Keywords:** Proteomics, Dental pulp, Supernumerary tooth, Mesiodens

## Abstract

**Objectives:**

To obtain and compare the protein profiles of supernumerary and normal permanent dental pulp tissues.

**Materials and methods:**

Dental pulp tissues were obtained from supernumerary and normal permanent teeth. Proteins were extracted and analyzed by liquid chromatography-tandem mass spectrometry (LC/MS-MS). Protein identification and quantification from MS data was performed with MaxQuant. Statistical analysis was conducted using Metaboanalyst to identify differentially expressed proteins (DEPs) (P-value < 0.05, fold-change > 2). Gene Ontology enrichment analyses were performed with gProfiler.

**Results:**

A total of 3,534 proteins were found in normal dental pulp tissue and 1,093 in supernumerary dental pulp tissue, with 174 DEPs between the two groups. This analysis revealed similar functional characteristics in terms of cellular component organization, cell differentiation, developmental process, and response to stimulus, alongside exclusive functions unique to normal permanent dental pulp tissues such as healing, vascular development and cell death. Upon examination of DEPs, these proteins were associated with the processes of wound healing and apoptosis.

**Conclusions:**

This study provides a comprehensive understanding of the protein profile of dental pulp tissue, including the first such profiling of supernumerary permanent dental pulp. There are functional differences between the proteomic profiles of supernumerary and normal permanent dental pulp tissue, despite certain biological similarities between the two groups. Differences in protein expression were identified, and the identified DEPs were linked to the healing and apoptosis processes.

**Clinical relevance:**

This discovery enhances our knowledge of supernumerary and normal permanent pulp tissue, and serves as a valuable reference for future studies on supernumerary teeth.

**Supplementary Information:**

The online version contains supplementary material available at 10.1007/s00784-024-05698-z.

## Introduction

One potential developmental problem in children is the presence of supernumerary teeth, which are defined as any extra tooth formed in normal dentition [1]. The prevalence of supernumerary teeth in permanent dentition is about 0.1–3.8% [2–7]. The most common type is a mesiodens, located at the midline of the anterior maxillary region [2, 4, 5], which accounts for about 47–67% of all supernumerary teeth [8, 9]. Several theories have been proposed to explain the formation of supernumerary teeth, but the main etiology is still unclear [2, 4–7, 10]. Although supernumerary teeth have been categorized as developmental anomalies and the presence of these teeth was considered a sign of disorder, previous studies have reported using supernumerary teeth as a replacement for normal teeth and as alternative sources for tissue regeneration [7, 11, 12]. 

Dental pulp is the soft connective tissue located in the center of the tooth, which is surrounded by dental hard tissue [13, 14]. The major components of dental pulp are odontoblasts, fibroblasts, pulp stem cells, nerves, and blood vessels [15]. This pulp tissue is crucial in tooth formation and development, possesses the ability to regenerate, and plays a crucial role in a tooth’s defensive processes [16, 17].

Proteomics is the large-scale study of the interaction, structure, and function of proteins in order to understand the nature of a tissue, organ, organism, or disease [18]. As such, obtaining proteomics data for a particular tissue enhances our comprehension of its function and structure. To date, current literature on dental pulp tissue has focused mainly on the proteomic profile of normal permanent teeth [19–23]. The proteome of supernumerary dental pulp tissue has never been investigated, despite such teeth being far from fully understood. This study is interested in building a solid understanding of the differences between supernumerary dental pulp tissue and normal dental pulp tissue. We specifically aimed to determine the protein profile of supernumerary dental pulp tissue and compare it with that of normal dental pulp tissue.

## Methods

### Subject selection

The inclusion criteria were permanent teeth obtained from individuals who were not taking any medication and were in good health. The teeth were free of carious lesions and had normal pulpal diagnosis. Additionally, all teeth must be in either stage G or H of tooth development, according to Demirjian’s classification [24]. Stage G is defined as the stage in which the root canal walls are parallel and the apical ends are open, while stage H is defined as the stage in which the roots’ apexes are closed.

There were two groups in this study, with six subjects in each group (*n* = 6). For the supernumerary tooth group, pulp tissue samples were obtained from supernumerary permanent teeth located in the anterior maxillary region (mesiodens) of children aged less than 10 years. For normal permanent teeth, pulp tissue samples were obtained from third molars of adults aged less than 30 years. Teeth were extracted or surgically removed at the Dental Clinic of Faculty of Dentistry, Mahidol University. Patients and/or their legal guardians provided informed consent before tooth extraction. Exclusion criteria involved teeth with pathologies such as dental caries or pulpitis, patients in an unhealthy condition, and patients with syndromes such as cleft lip, cleft palate, Gardner syndrome, and cleidocranial dysostosis.

### Sample preparation

Subsequent to tooth extraction, the specimens were immediately preserved on ice (0 °C), then samples were immediately frozen with liquid nitrogen and stored at -80 °C until further experiment. On the experimental day, cementum and any residual soft tissue, including periodontal ligament, epithelial tissue, and apical papilla if presented, were mechanically removed from the teeth using periodontal curettes. The teeth were then cleansed with normal saline, and part of the enamel, dentin, and cementum were cut vertically with a high-speed bur under water coolant to avoid pulp tissue damage. Subsequently, each tooth was split using a dental elevator to expose the pulp, which was carefully removed using a sterile dental curette. The dental pulp tissue from each tooth was placed in a microcentrifuge tube and stored on ice (0 °C).

### Protein extraction and quantification

The protein extraction method was based on previous studies [19, 20]. Briefly, 100 µL of lysis buffer (Sigma-Aldrich, Missouri, United States) and 10 µL of Protease inhibitor cocktail (Roche, Mannheim, Germany) were added to the pulp tissue samples, after which each sample was ground and homogenized using a manual tissue homogenizer, with all handling at 0–4 °C. Next, the samples were vortexed and incubated on ice, followed by centrifugation at 20,000 g for 30 min at 4 °C. Then, the supernatants were collected and protein concentration was determined using a bicinchoninic acid protein assay kit according to the standard microplate procedure protocol [25].

### Mass spectrometry

Samples containing 5 µg of protein underwent in-solution digestion. Disulfide bonds were reduced using 5 mM dithiothreitol in 10 mM ammonium bicarbonate (AMBIC), and sulfhydryl groups were alkylated with 15 mM iodoacetamide at room temperature in the dark. Afterwards, sequencing grade porcine trypsin was employed for protein digestion. The resulting tryptic peptides were dried, resuspended in 0.1% formic acid, and subjected to nano-liquid chromatography tandem mass spectrometry (nanoLC-MS/MS). Specifically, the prepared samples were injected into an Ultimate3000 Nano/Capillary LC System (Thermo Scientific, UK) coupled to a ZenoTOF 7600 mass spectrometer (SCIEX, Framingham, MA, USA). Each sample underwent nanoLC/MS-MS analysis in triplicate [26].

### Data analysis

The acquired mass spectrometry raw data were analyzed with MaxQuant (version 2.4.2.0) for protein identification and quantification in individual samples. The Andromeda search engine was used to correlate MS/MS spectra to the UniProt Homo sapiens database [27]. MaxQuant’s standard label-free quantitation utilized specific settings, which required missed cleavages to be less than two, a 0.6 Dalton mass tolerance, trypsin digestion, fixed modification involving carbamidomethylation of cysteine, and variable modifications. Protein identification required peptides that have at least seven amino acid lengths and a minimum of one unique peptide. For downstream data analyses, proteins were considered identified if a minimum of two peptides were detected, including at least one unique peptide. Protein false discovery rate was set at 1%, estimated through reversed search sequences. The search allowed a maximum of five modifications per peptide, and referenced a FASTA file comprising proteins from the Homo sapiens proteome obtained from Uniprot. The software automatically incorporated potential contaminants from the contaminants.fasta file provided by MaxQuant into the search space [26]. The raw mass-spectrometric data were deposited in jPOST Repository https://repository.jpostdb.org (accession: PDX050860) [28].

The resulting MaxQuant ProteinGroups.txt file was imported into MetaboAnalyst 5.0 to perform orthogonal partial least squares discriminant analysis (OPLS-DA) and generate heatmaps and volcano plots. Proteins with statistically significant differential expression were identified based on fold-change (> 2.0) and P-value (< 0.05).

Gene Ontology (GO) annotation enrichment analysis including biological process, cellular component, and molecular function terms was performed using gProfiler (https://biit.cs.ut.ee/gprofiler/gost, version e110_eg57_p18_4b54a898).

## Result

Each group consisted of six pulp tissue samples from different individuals. In the supernumerary permanent tooth group, teeth were obtained from three girls and three boys between the ages of six and ten years old (mean age: 7.62 years). Two of the teeth in this group were in Demirjian’s stage G of tooth development, while the other four were in stage H of tooth development [24]. In the normal permanent tooth group, teeth were collected from three women and three men between the ages of 20 and 25 years old (mean age: 21.5 years). Out of the six teeth in this group, two were in stage G of tooth development, while four were in stage H, according to Demirjian’s classification [24]. Details on the collected teeth, including age and gender of the donors, are presented in Supplementary Table 1.

**Fig. 1:**
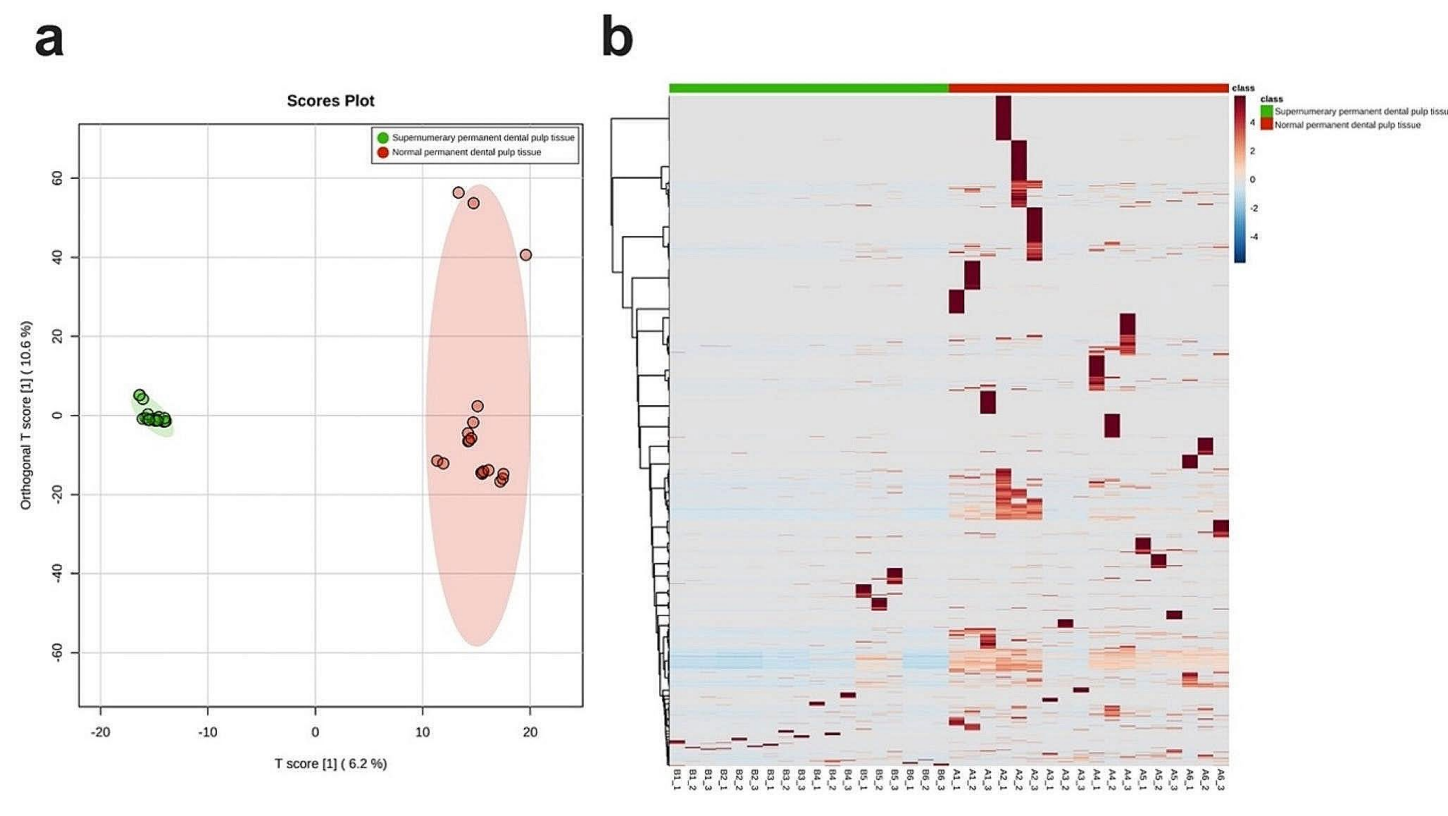
Distribution of protein abundances among samples and within replicates. **a**, Orthogonal partial least squares discriminant analysis **b**, Heatmaps

After obtaining the data from MaxQuant protein identification and quantitation, keratin proteins were excluded from the analysis to reduce the possibility of contamination from MS analysis [29]. As a results, a total of 3,534 proteins were identified in normal permanent dental pulp tissues (Supplementary Table 2), versus 1,093 identified in supernumerary permanent dental pulp tissues (Supplementary Table 3). Figure 1 illustrates the distribution and clustering of protein abundances across each group, between samples, and within replicates.

**Fig. 2:**
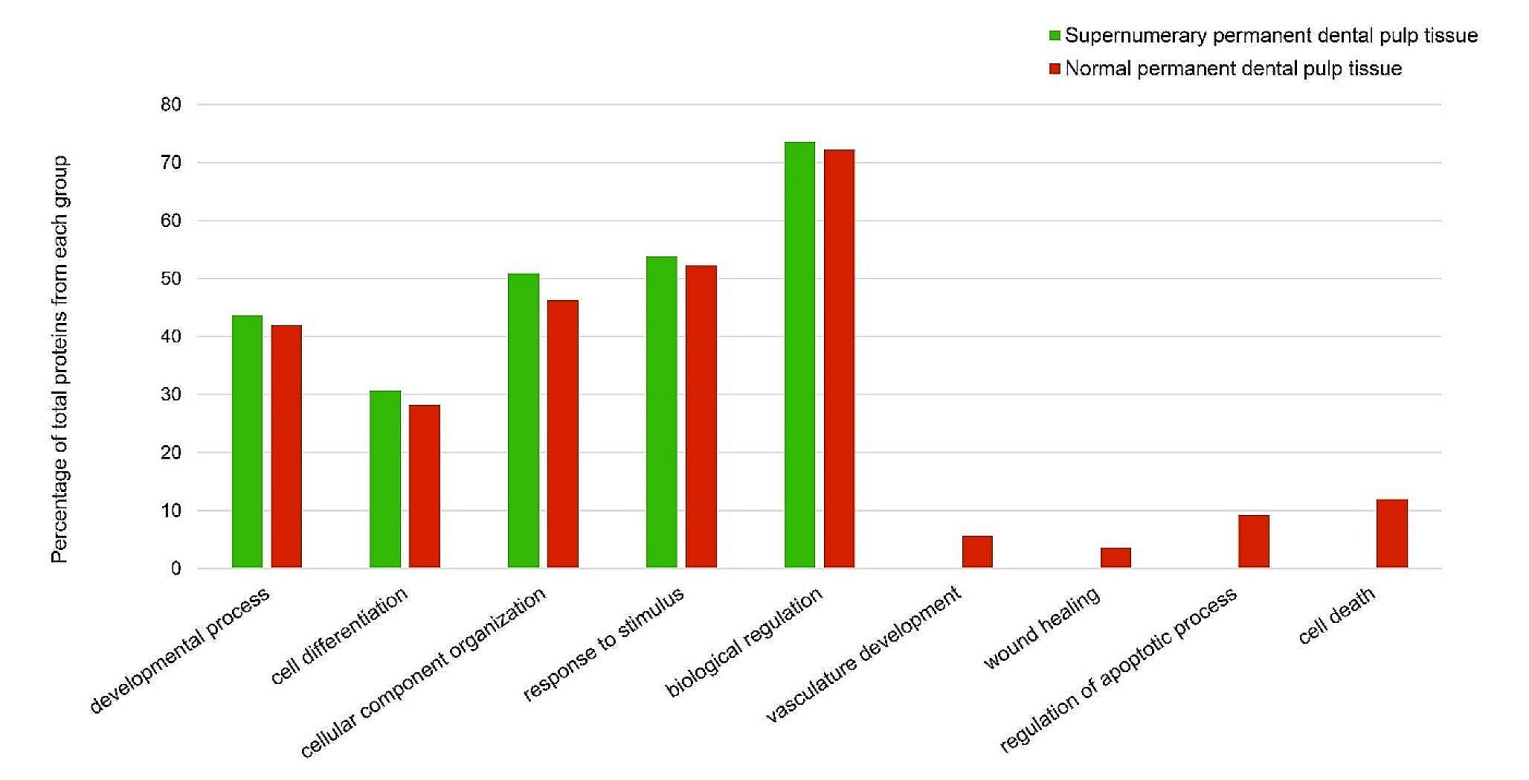
Comparison of functional characteristics of proteins found in supernumerary permanent and normal permanent dental pulp tissue

Protein abundance and functional characteristics were evaluated and compared across tissue conditions. Fig. S1 depicts the major biological process and molecular function terms associated with proteins detected in normal permanent dental pulp tissue. A comparison of the functional characteristics of supernumerary and normal permanent dental pulp tissue revealed several similarities and differences in the percentage of proteins found in each group. The two groups shared similar functions in terms of organization and development, differentiation, and response to stimulus. While different functions were primarily associated with apoptosis and cell death, healing, and vasculature development, as shown in Fig. 2.

**Fig. 3:**
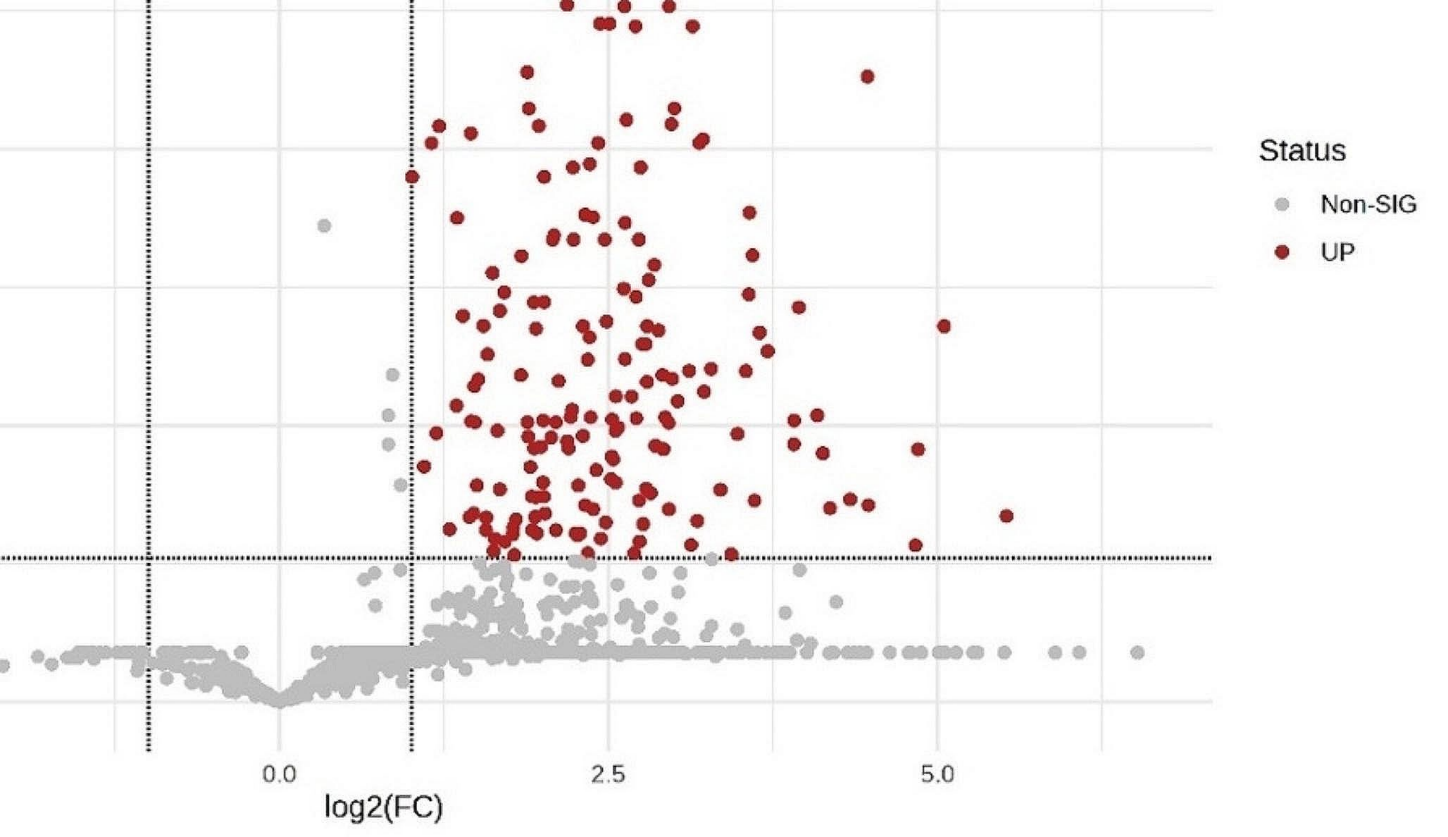
Volcano plot of –log10 (P-values) vs. log2 fold-change. Red dots indicate the 174 proteins upregulated in normal permanent dental pulp tissue. The vertical black lines correspond to 2.0 log2 fold-change (FC), up and down, and the horizontal black line represents an adjusted P-value of 0.05

**Fig. 4:**
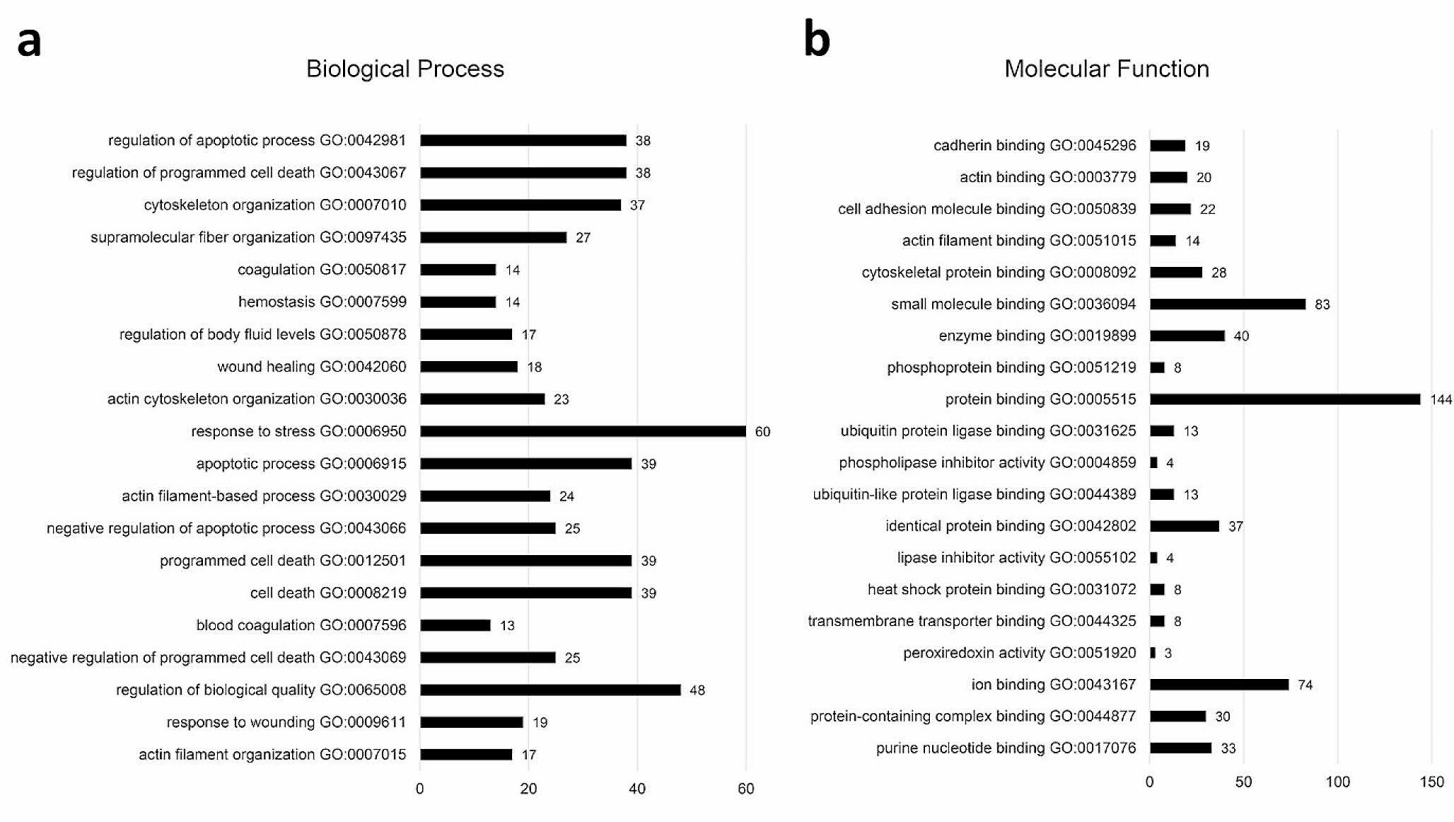
Top 20 most enriched GO **a** biological process and **b** molecular function terms among differentially expressed proteins. Numbers at the right represent the number of genes annotated with each term

Further analysis of differently expressed proteins (DEPs) found 174 proteins that were differently expressed between supernumerary permanent and normal permanent dental pulp tissue (P-value < 0.05 and fold-change > 2.0), as shown in Supplementary Tables 4 and Fig. 3. For DEPs, enriched biological process terms were mainly related to responses to stress and stimuli, wound healing, and regulation of cell death and apoptosis, while enriched molecular function terms related to the binding of proteins and ions (Fig. 4).

## Discussion

Supernumerary tooth is one of the most common developmental anomalies [1]. Presence of a supernumerary tooth may cause displacement or impaction of the adjacent permanent teeth [9]. Several theories have been postulated to explain the development and formation of supernumerary teeth, including their potential function as an alternative replacement of normal permanent teeth [12, 30]; however, the definitive etiology and main function of supernumerary teeth, especially at cellular and molecular levels, remains unclear [2, 4–7, 10].

Dental pulp tissue has been extensively investigated due to its crucial role in tooth development, maintenance, and function [16, 17]. However, the majority of current research, including proteomic profiling, has focused mainly on the dental pulp of normal permanent teeth [19–23]. Investigation into the structure and function of supernumerary tooth dental pulp is needed, as the proteomic profile cannot be assumed identical to that of a normal permanent tooth. This study is the first to investigate the proteomic profile of supernumerary dental pulp tissue and identify significantly different expression of proteins that may be related to supernumerary teeth.

Mesiodens of the permanent dentition were chosen as the representative of supernumerary permanent teeth in this study because that type is the most common supernumerary tooth and routinely needs to be removed due to complications [1–6]. Third molars were chosen as a representative of normal permanent teeth because they are typically maligned or impacted, indicating the need for removal [31]. The dental pulp tissue in this study was obtained from healthy subjects, and all teeth were sound. Each group contains an equal number of subjects from both sexes to minimize the impact of sex differences on the functions and response processes of pulp tissue [2, 32]. Furthermore, as the stage of tooth development has an impact on the biological functions of dental pulp, to minimize these effects, the teeth obtained from each group were in a similar stage of tooth development, with two teeth in each group in stage G and the other four teeth in each group in stage H of tooth development, according to Demirjian’s classification [24].

To reduce epithelial cell contamination, all remaining soft tissue and cementum were mechanically removed with a periodontal curette. Although complete elimination of non-specific protein contamination may not be possible, these contaminants can be reduced. Keratin proteins were excluded from the analysis for the reason that they are commonly regarded as contaminants [29].

OPLS-DA revealed distinct intergroup differences along with intragroup sample homogeneity, reflected in the heatmap of protein expression intensities. The distinct difference revealed by this analysis suggests that specific proteins are expressed differently in supernumerary and normal permanent dental pulp tissues.

Overall, 3,534 proteins were detected in normal permanent dental pulp tissues (Supplementary Table 2), which is within the range of 66 to 4,332 proteins detected in previous studies [19–23], (Supplementary Table 5). The wide variation in total identified proteins in dental pulp tissue may be due to different studies employing different methods of protein extraction, labeling, and separation; mass spectrometry techniques; and criteria for protein identification [19–23]. Despite differences in total detected proteins, GO enrichment analysis based on biological process terms in the present study highlighted similar functions as compared to previous studies of normal permanent dental pulp tissue (Fig. S1) [19–21, 23]. For supernumerary permanent teeth, a total of 1,093 proteins were detected (Supplementary Table 3). Interestingly, according to previous reports and this study, there have only been roughly 66 − 4,332 identified proteins in dental pulp tissue thus far (Supplementary Table 5) [19–23]. This is a relatively low number when compared to the number of proteins found in other tissue or in a single cell that have been previously reported [33, 34]. It is still unknown why there are so few proteins found in dental pulp; more research is required to solve this mystery. This could, however, also draw attention to how distinctive and special dental pulp tissue is.

Comparison of the functional characteristics of supernumerary and normal permanent dental pulp tissue revealed that dental pulp tissue from both groups displayed similar functions in terms of cellular component organization, biogenesis, cell differentiation, and developmental processes. These are the major functions of dental pulp that play a crucial role in tooth development, maturation, and the regenerative process [16, 17]. Moreover, similar functions of supernumerary and normal permanent dental pulp tissue were also found in terms of response to stimulus, an important defense mechanism that protects the pulp from any harmful stimulus in order to maintain the vitality of the tooth [13].

While supernumerary and normal permanent dental pulp tissue shared several functional characteristics, differences in protein functions were also observed. Differences were found in the biological processes of wound healing and vasculature development, which clearly are the important mechanisms for tissue regeneration [35]. These functions were only detected in the dental pulp of a normal permanent tooth. Moreover, the function of cell death and regulation of apoptosis process, which normally occur as part of the regenerative process and wound healing of dental pulp [36, 37], were only identified in normal permanent dental pulp tissue.

Furthermore, comparative analysis of DEPs between supernumerary permanent and normal permanent dental pulp tissue identified 174 proteins that were significantly differentially expressed (Supplementary Table 4). GO enrichment analysis of the DEPs revealed enrichment of molecular functions related to protein and ion binding. Differences in functional characteristics between supernumerary and normal permanent dental pulp tissue were also confirmed by DEPs. The biological functions of DEPs were mainly related to responses to stress and stimuli, wound healing, and regulation of cell death and apoptosis. Interestingly, it has been proposed that the development of a supernumerary tooth is significantly influenced by control of the apoptotic process [38, 39].

Possible limitations of this study that may impact its findings primarily concern the small sample size, which is a consequence of the limited number of patients with mesiodens available throughout the study period; this is due to the prevalence of mesiodens in permanent dentition being less than 3.8% [2–7]. Additionally, the study encountered challenges related to age differences between the two groups, as mesiodens complications typically require removal in children during the mixed dentition phase, whereas third molar removal is required during late adolescence or adulthood. Consequently, it is impractical to collect both supernumerary permanent and normal permanent dental pulp tissues from donors of the same age, and also from the same donor. Despite the difference in chronological age between the two groups of donors, our study mainly focused on dental age, specifically referring to the stage of tooth formation. Both groups exhibited similar stages of tooth development, categorized as tooth development stages G and H according to Demirjian’s classification [24]. In spite of these limitations, the collected samples are sufficient for statistical analysis and screening purposes in this initial exploration of the characteristics of supernumerary permanent dental pulp tissue. Future studies should validate DEPs to confirm the findings and include a larger sample size for more specific investigations.

In conclusion, this study provides a comprehensive understanding of the proteomic profile of permanent dental pulp tissue, and is furthermore the first to investigate the protein profile of supernumerary permanent dental pulp tissue. Dental pulp tissue of normal permanent and supernumerary permanent teeth displayed similar functional characteristics in terms of cellular component organization, cell differentiation, developmental process, and response to stimulus. While the functional characteristics of healing, vascular development and cell death were only detected in normal permanent dental pulp. Differences in protein expression were identified between supernumerary and normal dental pulp tissue of permanent teeth, these proteins were found to be associated with the processes of wound healing and apoptosis.

### Electronic supplementary material

Below is the link to the electronic supplementary material.

Supplementary Material 1

Supplementary Material 2

## Data Availability

Data is provided within the manuscript or supplementary information and the raw mass-spectrometric data were deposited in jPOST Repository https://repository.jpostdb.org (accession: PDX050860).
