# Supplementary figures and images for: Comparative proteomic analysis of dental pulp from supernumerary and normal permanent teeth
Source: Clin Oral Investig. 2024 May 17;28(6):321. doi: 10.1007/s00784-024-05698-z (PMC11101566; doi:10.1007/s00784-024-05698-z)

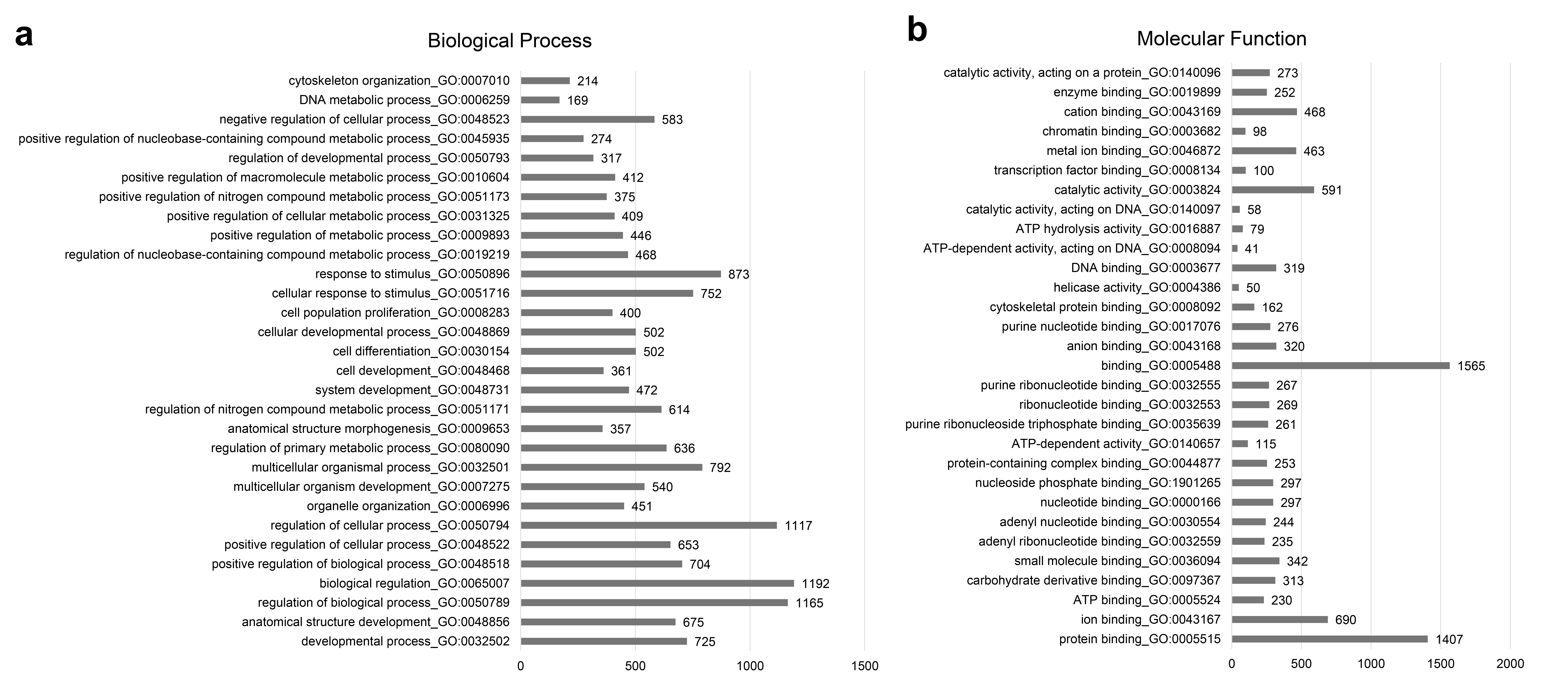

Supplement: Supplementary file 1 — Supplementary Material 1 [file 784_2024_5698_MOESM1_ESM.jpg]
